# NSAID choice: lessons from PRECISION

**DOI:** 10.18632/aging.101930

**Published:** 2019-04-29

**Authors:** Grant W. Reed, Steven E. Nissen

**Affiliations:** 1 Department of Cardiovascular Medicine, Heart and Vascular Institute, Cleveland Clinic, Cleveland, OH 44195, USA

**Keywords:** NSAID, PRECISION, celecoxib, aspirin, cardiovascular events

Nonsteroidal anti-inflammatory drugs (NSAIDs) have important adverse effects including the potential to increase cardiovascular, gastrointestinal, and renal events. Despite these risks, NSAIDs are among the most commonly administered medications in the world, administered to approximately 1 in 8 US adults on a regular basis [1]. Due to heightened cardiovascular risks [2], the selective cyclooxygenase (COX)-2 inhibitors rolfecoxib (Viox) and was removed from the US market in 2004. While selective COX-2 inhibitor celecoxib (Celebrex) was allowed to remain on the market, the Food and Drug Administration (FDA) mandated a trial to study the cardiovascular safety of celecoxib in comparison with non-selective COX inhibitors ie. drugs that inhibit both COX-1 and COX-2.

The Prospective Randomized Evaluation of Celecoxib Integrated Safety vs. Ibuprofen or Naproxen (PRECISION) trial was conducted to address this regulatory concern [3]. PRECISION randomized 24,081 patients with NSAID-dependent osteoarthritis (OA) or rheumatoid arthritis (RA) at elevated cardiovascular risk to moderate-dose celecoxib 100-200 mg twice daily (BID), ibuprofen 600-800 mg three times daily (TID), or naproxen 375-500 mg BID for symptom relief. The results of PRECISION demonstrated comparable cardiovascular safety for celecoxib. In an intention-to-treat (ITT) analysis, celecoxib was non-inferior to naproxen and ibuprofen for the primary composite outcome of cardiovascular death, nonfatal myocardial infarction, or nonfatal stroke during a mean follow-up of 34.1 months (event rates of 2.3% vs 2.5% vs 2.7% respectively; P<0.001 for non-inferiority for comparisons to either NSAID). Moreover, celecoxib had certain advantages, as there were fewer gastrointestinal events with celecoxib (1.1%) compared to either naproxen (1.5%) (P=0.01) or ibuprofen (1.6%) (P=0.002), and fewer renal events with celecoxib (0.7%) compared to ibuprofen (1.1%) (P=0.004) [3].

The PRECISION trial results should reassure providers that celecoxib is unlike rofecoxib - moderate-dose celecoxib showed similar cardiovascular safety to ibuprofen or naproxen. However, the question remains - are there patient subgroups that benefit from a specific NSAIDs more than others?

A common clinical issue is the management of NSAID-dependent patients also taking aspirin. The rationale for this concern based on *in vitro* studies that suggest NSAIDs may decrease the anti-platelet efficacy of aspirin via inhibition of prostanoid synthesis and by blocking aspirin’s ability to inhibit COX-1, which is required for platelet inhibition [4]. However, the clinical significance of this theoretical issue has not been demonstrated, and high-quality, outcome data on concomitant NSAID and aspirin use have not been previously available. To address this evidence gap, the PRECISION trial pre-specified an analysis of outcomes stratified by aspirin use – the findings of which are both surprising and informative to patient care.

The PRECISION Aspirin substudy results demonstrate that while on study drug, patients who were not on aspirin had *better* overall safety outcomes on celecoxib than ibuprofen or naproxen [5]. Specifically, celecoxib associated with *less* composite cardiovascular, gastrointestinal, and renal events, as well as all components of this composite (Kaplan-Meier P<0.01 for all comparisons). The addition of aspirin appeared to attenuate the safety advantage of celecoxib, as though celecoxib still associated with fewer composite safety events than either ibuprofen or naproxen (P<0.0001), this was driven by fewer GI events (P=0.004), and there was no difference in CV or renal events between agents [5].

From these results, there are several important messages. First, aspirin can be taken safely with either celecoxib, ibuprofen, or naproxen as cardiovascular safety is equivalent among the agents. However, celecoxib may have certain advantages, since it is associated with fewer GI events among aspirin users. Further, if aspirin is not needed for secondary prevention, it should be avoided. In patients without an indication for aspirin, celecoxib has better overall and cardiovascular safety, and based on these data, should be considered the NSAID of choice.

It is reasonable to conclude from PRECISION that the hypothesis that selective COX-2 inhibitors as a drug class inherently worsen cardiovascular outcomes is not supported by clinical data. Indeed, the addition of aspirin (a selective COX-1 inhibitor) to celecoxib (a selective COX-2 inhibitor) actually slightly *worsened* cardiovascular outcomes in PRECISION, and the best cardiovascular outcomes were observed when celecoxib was taken alone. The medical community should be encouraged to embrace these results rather than hold on to findings from *in vitro* and smaller observational studies [6,7], as PRECISION is the highest-quality clinical outcome data of selective COX-2 and combined COX-1/COX-2 inhibitors to date.

We have made considerable progress in this field over the past decade, which has been accelerated by PRECISION. Nonetheless, several important questions regarding the long-term safety of NSAIDs remain: Are cardiovascular outcomes affected by NSAID dose? Is there a difference in safety in patients without or with established coronary artery disease (i.e. primary or secondary prevention populations)? Are there other specific risk factors that may lend to better outcomes with celecoxib compared to the other NSAIDs? Through addressing these questions, we will continue to improve outcomes and patient care in the aging population.
